# Alcohol Research and Social Policy: An Overview

**Published:** 1996

**Authors:** Enoch Gordis

**Affiliations:** Enoch Gordis, M.D., is director of the National Institute on Alcohol Abuse and Alcoholism, Bethesda, Maryland

**Keywords:** public policy on AOD, AOD consumption, research, minimum drinking age laws, warning label, alcoholic beverage, moderate AOD use, therapeutic drug effect, United States, federal government, government agency

## Abstract

Science can facilitate the task of choosing among complex social policies, although it rarely serves as the only basis for policy development. Science’s role in policy formation can be decisive when public support already exists, as with the passage of the Federal Uniform Drinking Age Act. Science can assess a policy after it has been implemented, as in the scientific evaluation of the health warning labels on alcoholic beverage containers. In addition, science can investigate the short- and long-term benefits and risks of areas where the development of policies is likely. An example is the current scientific examination of the tradeoffs involved in moderate alcohol consumption.

By its very nature, the phenomenon of alcohol consumption creates many opportunities for social policy development and implementation. Alcohol is a part of our culture and is widely used in many social and ceremonial activities. Its manufacture and sale produce revenue for the government, through taxes, and income for citizens, through business profits and employment. For some people, moderate alcohol use also may provide health benefits.

The use of alcohol also has negative implications for the social, economic, and health status of both those who use it and society at large. About 14 million Americans meet medical diagnostic criteria for alcohol abuse or alcoholism (Grant et al. 1991). The consequences of alcohol abuse and dependence cost the Nation an estimated $99 billion (Rice 1993) and 100,000 deaths each year (National Institute on Alcohol Abuse and Alcoholism [NIAAA] 1993). Alcohol misuse is estimated to be involved in about one-half to two-thirds of homicides, one-fourth to almost one-half of serious assaults, one-third of suicides, and more than one-fourth of rapes (Martin 1992). In addition, 20 to 40 percent of patients in urban hospital beds have alcohol problems, regardless of the conditions for which they were initially hospitalized (Moore et al. 1989).

Alcohol sales and consumption are regulated for economic, health, and social purposes. To achieve these purposes, many agencies at the Federal, State, and local levels develop and carry out policies that affect the distribution and use of alcohol. Choosing among policies to accomplish the greater good is not easy, and one policy always runs the risk of being at cross-purposes with another. Science can help make the task of choosing among policies more rational.

This article provides an overview of how alcohol policies are developed in the United States and examines the role of science in the development of three specific policies: raising the minimum legal drinking age (MLDA), requiring health warning labels on alcoholic beverage containers, and formulating recommendations concerning the risks and benefits of moderate drinking.

## What Is Alcohol Policy in the United States?

U.S. alcohol policies generally fall into two categories: (1) those intended to influence individual drinking practices and (2) those aimed at regulating the supply of alcoholic beverages.

Policies to influence individual drinking patterns have included publicly financed information and education programs, as well as State and local laws establishing penalties for drinking and driving. One example of a policy designed to increase public awareness of several specific health risks of alcohol consumption is the requirement for a health warning label on alcoholic beverage containers sold in the United States. Another policy is the requirement of individual States for mandatory sentencing of persons convicted of drinking and driving offenses.

Some policies are designed to limit access to alcohol. Policies in this category include raising the MLDA; restricting the number, location, and business hours of alcoholic beverage sales outlets; and prohibiting the promotion of alcoholic beverages on college campuses. In addition “dram shop” laws influence the drinking environment to help prevent adverse alcohol-related consequences. These laws hold drinking establishments and, in some cases, private hosts liable for alcohol-related damages caused by a person to whom they have served alcoholic beverages.

## Who Influences Policy Development?

Alcohol-related policy development in the United States is complex. Federal, State, and local governments are involved in regulation, and nongovernmental bodies, such as citizen and industry organizations, seek to influence policy directions.

### Federal Agencies

The major responsibility for policy determination at the Federal level rests with the U.S. Congress, which passes laws; the President, who signs laws; and the U.S. Supreme Court, which interprets laws. The responsibility for implementing Federal alcohol policies resides in several different Executive branch departments and agencies. For example, health warning labels on alcoholic beverage containers are regulated by the Department of the Treasury and by the Federal Trade Commission. The Treasury Department also is responsible for administering Federal alcohol-related tax policies and for collecting the associated revenues. Dietary guidelines containing recommendations on alcohol consumption are the joint responsibility of the Departments of Health and Human Services (DHHS) and the United States Department of Agriculture (USDA). The USDA also is responsible for policies that affect agricultural production, including the production of grain and grape crops that are used in alcoholic beverages.

The Department of Transportation is concerned with alcohol-related transportation issues, including highway safety and the use of alcoholic beverages by airplane pilots, railway workers, and ship personnel. Alcohol prevention polices are under the purview of DHHS and the Departments of Transportation (DOT) and Education. Alcohol treatment policies, including policies on health care reimbursement, are developed by DHHS and the Departments of Veterans Affairs (VA) and Defense (DOD), as well as by the Social Security Administration.

Research on alcohol-related issues is undertaken by DHHS, the VA, DOD, and DOT; however, the primary agency devoted to alcohol research is the National Institute on Alcohol Abuse and Alcoholism (NIAAA), one of the National Institutes of Health located within DHHS.

As is apparent, policy and program coordination within this Federal structure is an ongoing necessity. Attempts have been made to establish standing interagency coordinating committees to promote consistency in Federal policy development. These committees, comprised of representatives of all Executive branch entities with alcohol-related responsibilities meeting on a regular basis, proved less effective than hoped. Not every department has a role to play in the development or implementation of every policy and time spent by a large number of agencies on issues clearly not within their jurisdictions proved wasteful in terms of both time and programmatic accomplishment. Instead, coordination among Departments and agencies is effectively handled on a case-by-case basis, with relevant parties coming together as necessary. Such was the case when the Bureau of Alcohol, Tobacco and Firearms (BATF), an agency of the Treasury Department, was requested to approve a “hang tag” for wine bottles that proclaimed certain health benefits associated with moderate drinking (the so-called “French Paradox”). In considering this request, the BATF, which had primary policy jurisdiction in this issue, consulted with the FTC and the NIAAA for policy (FTC) and scientific (NIAAA) information relevant to the issue. (The request to approve the “hang tag” subsequently was withdrawn.)

**Federal Government Departments’ Roles in Alcohol Policy**

**Health and Human Services (DHHS)**

Research, prevention, and treatment
Medicare/medicaid

**Defense and Veterans’ Affairs**

Research and treatment for active-duty personnel, dependents, and eligible veterans

**Treasury**

Tax policies
Regulation of advertising claims
Alcohol production

**Education**

Alcohol prevention programs

**Agriculture (with DHHS)**

Dietary guidelines

### State Agencies

The 21st amendment to the U.S. Constitution, which repealed Prohibition, also ceded to the States the authority to regulate many aspects of commerce with respect to alcoholic beverages. The States, therefore, individually enact policies governing how alcohol is sold, who may drink alcohol and where, and how alcohol may be advertised. They set penalties for the inappropriate use of alcohol and also may impose taxes on alcoholic beverages. Not only is each State free to develop its own policies, but many States permit local governments to establish their own policies concerning alcoholic beverage use. Thus “wet counties,” which permit the sale of alcoholic beverages, may border “dry counties,” which prohibit some or all forms of alcoholic beverage distribution. (State policy actions may be superseded by Federal actions. See the discussion below on interactions among multiple levels.)

**States’ and Local Governments’ Roles in Alcohol Policy**

States individually enact policies governing the following:

- How alcohol is sold
- Who may drink alcohol and where
- Limits and penalties for the inappropriate use of alcohol
- How and where alcohol is advertised within the State.

States’ policy actions may be superseded by Federal actions.

States may share policy development with local governments.

### The Private Sector

Numerous nongovernmental entities influence policy development. These include national organizations, such as the National Council on Alcoholism and Drug Dependence and Mothers Against Drunk Driving; members of the alcoholic beverage industry, such as producers (distillers, vintners, brewers) and distributors; and State, county, and municipal citizen advocacy and advisory groups. Private sector groups ensure nongovernmental participation in essentially governmental processes and provide policymakers with a variety of views supporting or opposing potential and existing policies. Strong advocates for certain policies can make a difference in whether or not a policy is actually adopted. For example, a coalition of many private sector groups, along with scientific and governmental groups, led the fight in 1970 to establish the NIAAA. Although private sector groups can be active participants in policy development, the promulgation and implementation of policies generally is carried out by government. (One exception has been in the area of alcohol advertising policies, which have been voluntarily developed, implemented, and monitored for compliance by the alcoholic beverage industry.)

Participation by such nongovernmental entities can make the policy formulation process quite complex. At times, what is advocated by one set of private sector groups may conflict with the views of other private sector groups. For example, as noted above, advertising has been voluntarily regulated by the alcoholic beverage industry. A current policy-related discussion concerns whether governmental restrictions should exist on the advertising of alcoholic beverages, and if so, what they should be. Private sector groups representing a variety of opinions both for and against restrictions are actively engaged in discussions in a variety of forums. Ultimately, these discussions, along with public opinion that they may generate and relevant scientific data, will form the basis for government action to establish or to decline to establish new policies governing alcohol advertising.

### Interactions Among Multiple Levels

Although the U.S. Constitution gives the States the authority to regulate the distribution, sale, and use of alcoholic beverages, the States must meet Federal criteria in this process. For example, alcoholic beverage containers are required by Federal law to have health warning labels. The health warning messages, as well as the design and placement of the health warning label, are set forth in Federal law and regulation.

State laws also are subject to constitutional review by the Federal courts. In one recent instance, a group of industry representatives challenged a Rhode Island law prohibiting the advertising of alcoholic beverage prices, claiming that the ban infringed on their freedom of speech. In defending the constitutionality of this law before the U.S. Supreme Court, Rhode Island argued that the authority the 21st amendment to the U.S. Constitution grants to the States to regulate commerce in alcoholic beverages supersedes, for those engaged in the sale of alcohol, the 1st amendment guarantee of free speech. In its decision on the matter, *44 Liquormart v. Rhode Island* (64 U.S.L.W. 4313[1996]), the Court rejected this argument and struck down the restriction on price advertising on free speech grounds. How this ruling will ultimately affect other forms of advertising for alcoholic beverages remains to be seen.

Finally, the Federal Government can use the financial relationship it has with the States to foster the adoption of alcohol-related policies. A prime example of this interaction is the passage of the 1984 Federal Uniform Drinking Age Act, which tied the granting of monies from the Federal Highway Trust Fund to State passage of laws establishing the minimum legal drinking age (MLDA) at 21 years. The National Highway System Designation Act of 1995 provides a similar incentive to the States to achieve a national “zero-tolerance” policy. Under this legislation, States must enact and enforce a law that considers a driver under the age of 21 with a blood alcohol concentration (BAC) of 0.02 percent or greater to be legally intoxicated. Any State that does not comply by 1999 will begin losing a portion of its Federal Highway Trust Fund share.

## What Is the Role of Science in Policy Development?

Embodied in laws and regulations, public policies are usually developed when public concern over a problem has reached a critical level. Science can play a decisive role in policy development when public support already exists, but rarely does science serve as the only basis on which policies are developed. A mix of economic, cultural, religious, and political pressures is often more important to the process than scientific evidence for or against a particular policy.

How can science help? Occasionally, strong evidence for a particular policy converges with sufficient public support to effect a policy. Such is the case with the passage in 1984 of the Federal Uniform Drinking Age Act and the more recent passage of Federal legislation mandating zero-tolerance policies in the States. In many cases, however, deciding which policies to implement is like deciding whom to marry—often one must make a decision before all the evidence is in. In these instances, science can still play a role by assessing a policy after it has been implemented. One example is the scientific evaluation of the health warning labels on alcoholic beverage containers (discussed below). Science also can investigate the short-and long-term benefits and risks of potential policies. The current scientific examination of the benefits and risks of moderate alcohol drinking over the life span is an example of how science can contribute to the public dialog around issues that have the potential to generate policy choices.

## Raising the Minimum Drinking Age and Zero Tolerance: Using Science as a Resource in Policy Development

Scientific evidence strongly influenced Federal efforts to encourage all States to adopt an MLDA of 21 years, as well as subsequent policies to encourage the States to adopt zero-tolerance policies for underage drinkers.

The early 1970’s (i.e., 1970 to 1975) brought about a trend among the States to lower the minimum age at which a person could purchase or possess alcohol. By the mid-1970’s, highway safety statistics began to show marked increases in alcohol-related traffic deaths, particularly among young people ages 16 to 24, who were heavily overrepresented among those who were dying on the highways. Public concern also was raised over the lack of consistency among State MLDA laws, which during the mid- to late 1970’s ranged from 18 to 21. In general, the concern was that inconsistencies in the States’ laws created incentives for youth to cross State borders to procure alcohol in jurisdictions with lower MLDA’s, thereby increasing their risks for alcohol-related injury and death. Beginning in the mid-1970’s, many States began to raise their MLDA’s. Studies of the impact of these changes found that raising the MLDA reduced alcohol-related traffic crashes among young people affected by the law. Moreover, evidence indicated that these effects persisted over several years (O’Malley and Wagenaar 1991; Wagenaar 1993).

In 1984 the convergence of significant public concern and a firm body of scientific evidence resulted in the passage of the Federal Uniform Drinking Age Act, which called for all States to raise the MLDA to 21. Compliance by all 50 States was achieved in 1988. Research continues to show that an MLDA of 21 prevents drinking-and-driving-related crashes and fatalities among drivers under 21.

More recently, science is serving as a resource in the national policy debate concerning underage drinking and alcohol-related traffic fatalities among youth. Continuing concern about these issues has resulted in growing support for the passage of zero-tolerance laws. These laws specify a maximum legal BAC of 0.02 or lower for drivers younger than age 21. States that enacted zero-tolerance laws experienced an average 20-percent reduction in fatal single-vehicle nighttime (SVN) crashes, compared with States that did not lower the legal BAC for underage drinkers. According to researchers, if all States adopted these BAC limits for drivers ages 15 to 20, at least 375 fatal SVN crashes would be prevented each year (Hingson 1994). Continued public concern and support for strict measures to reduce underage alcohol use, coupled with the clear scientific evidence of the success of the MLDA law, has resulted in the passage or consideration of zero-tolerance laws in a number of States, and as noted above, the passage of the Federal National Highway System Designation Act.

### Health Warning Labels: Science Can Help After Implementation

The coalescing of public support, rather than scientific evidence, resulted in the passage of legislation requiring health warning labels on alcoholic beverage containers. Science, however, has been able to provide valuable information on how effective this particular policy has been both as a public information tool and as an instrument for changing behavior.

In 1988 legislation was enacted requiring all alcoholic beverages (i.e., beer, wine, and distilled spirits) bottled on or after November 18, 1989, to carry a label warning the public of several significant health risks associated with alcohol use: birth defects (if alcohol is consumed during pregnancy), impairment of ability to drive a car or operate machinery, and “health problems.” The issue of an alcohol health warning label had been debated for nearly two decades, beginning in 1972 with bills introduced in the U.S. Senate that would have required a health warning label on distilled spirits.

A 1980 report issued jointly by the Department of the Treasury and DHHS summarized then-current scientific knowledge regarding birth defects and a wide range of other health hazards associated with alcohol consumption. This report noted that available evidence with regard to cigarette and saccharin health warning labels suggested that to be effective, health warning labels must be specific as to the risks that might be incurred. Because scientific evidence regarding the health risks of alcoholic beverages was limited, the report concluded that requiring such labels on alcoholic beverage containers was premature.

By 1986, as a result of continuing public pressure, Federal legislation requiring health warning labels once more was introduced in both houses of Congress. As a part of the DHHS development of policy options and recommendations with respect to this legislation, NIAAA initiated a review of scientific literature on the effectiveness of health warning labels in communicating risk and changing behavior. The review considered scientific evidence of the effectiveness of health warning labels for a variety of consumer products, such as tobacco and food, as well as for alcoholic beverages in countries other than the United States. Although supportive of alcohol health warning labels in principle, the conclusions of this review were more suggestive than definite. Based on the scientific evidence, the DHHS recommendation to Congress did not express support for health warning labels.

As happens often in the case of public policy debate, the public’s *belief* in the value of health warning labels made the difference. For example, supporters of the measure argued that because health warning labels were required for bubble bath, aspirin, and other widely used products, they also should be required for one of America’s most widely used and abused drugs—alcohol. The intrinsic plausibility of such arguments was beyond dispute, despite the scant scientific evidence for the actual effectiveness of such labels.

The final passage of warning-label legislation provided science with the opportunity to study the influence of the labels on public knowledge, attitudes, and behaviors with regard to alcohol use. Although the warning labels do appear to have increased public awareness of the three alcohol-related health risks described on the labels, they do not appear to have had a major influence on changing behavior (Hilton 1993). The above conclusions, provided by science after the implementation of the policy, are available to help policymakers as they consider future options to reduce the consequences of alcoholic beverage consumption.

### Science Weighs Moderate Alcohol Consumption Before Policy Is Implemented

Many studies show that moderate alcohol consumption has some health benefits—specifically, it reduces the risk of cardiovascular disease. In postmenopausal women, low-level alcohol consumption also may enhance estrogen production, which in turn may provide protection from osteoporosis as well as from coronary heart disease (Tivis and Gavaler 1994). Yet, for some groups of people, moderate alcohol consumption also can increase the risk for alcohol-related health problems, including adverse fetal effects and traffic crashes. Therefore, not just the quantity and frequency but also the timing of alcohol consumption may be important in weighing the risks and benefits of moderate drinking.

For example, although research has demonstrated cardioprotective benefits from moderate alcohol consumption, this benefit accrues from drinking over the life span and therefore mainly affects older drinkers. On the other hand, moderate drinking by younger persons, who generally are not at risk for heart disease, places them at increased risk for alcohol-related traffic fatalities. Members of the alcoholic beverage industry frequently have requested Federal Government approval of labeling claiming the health benefits of moderate drinking. Such a policy, if implemented, not only might help reduce deaths from coronary disease but also might increase the risk of alcohol-related traffic fatalities for younger persons.

Should moderate alcohol consumption be encouraged or discouraged? By describing the health risks and benefits associated with different levels of alcohol consumption over the life span, science can provide critical information to help consumers make sensible decisions about drinking. We need to know much more about the tradeoffs involved in moderate alcohol consumption, including the following information:

- A fuller understanding of who is at risk, and for what problems
- Who stands to benefit from moderate alcohol consumption and in what ways
- How patterns of risks and benefits vary over the life span for various segments of the population.

## Conclusion

Alcohol research has the potential to assist policy development and decisionmaking. As our research base on alcohol-related problems expands, public policy advocates, policymakers, and consumers will be better able to understand the potential impacts of their actions. As a result, they will be able to plan programs and implement public policy strategies that have the greatest chance to prevent and reduce alcohol-related problems and to improve overall health outcomes.
